# Draft Genome Sequence of *Enterobacter cloacae* Strain JD6301

**DOI:** 10.1128/genomeA.00381-14

**Published:** 2014-05-29

**Authors:** Jessica G. Wilson, William T. French, Anna Lipzen, Joel Martin, Wendy Schackwitz, Tanja Woyke, Nicole Shapiro, James W. Bullard, Franklin R. Champlin, Janet R. Donaldson

**Affiliations:** a Department of Biological Sciences, Mississippi State University, Mississippi State, Mississippi, USA; b Renewable Fuels and Chemicals Laboratory, Dave C. Swalm School of Chemical Engineering, Mississippi State University, Mississippi State, Mississippi, USA; c DOE Joint Genome Institute, Walnut Creek, California, USA; d Schools of Biomedical Sciences and Forensic Sciences, Oklahoma State University for Health Sciences, Tulsa, Oklahoma, USA

## Abstract

*Enterobacter cloacae* strain JD6301 was isolated from a mixed culture with wastewater collected from a municipal treatment facility and oleaginous microorganisms. A draft genome sequence of this organism indicates that it has a genome size of 4,772,910 bp, an average G+C content of 53%, and 4,509 protein-coding genes.

## GENOME ANNOUNCEMENT

*Enterobacter* is a genus in the family *Enterobacteriaceae*. This family is composed of Gram-negative rods, which can be found in many different environments, ranging from soil to humans. Many strains of *Enterobacter* are known to be resistant to an array of antibiotics, and some are considered nosocomial pathogens (1). Here, we report the genome sequence of the novel *Enterobacter cloacae* strain JD6301. This novel strain was isolated from a coculture of wastewater collected from a municipal treatment facility and oleaginous bacteria. The bacterial strain was isolated by subsequent dilutions into fresh medium and finally by culturing onto nutrient agar under aerobic conditions at 30°C. The bacteria were found to grow under a wide variety of conditions, including temperatures ranging from 28 to 39°C, and they were also found to tolerate slightly acidic conditions (pH 4.5). On blood agar, JD6301 exhibits alpha-hemolysis. MacConkey and eosin methylene blue agar confirm that it is capable of utilizing lactose. It is oxidase negative and produces gas in triple sugar iron agar. The most unique feature of JD6301 is its ability to form inclusion bodies, which has not been characterized for this species. The lipid weight of this strain was found to constitute approximately 50% of the total cellular weight, suggesting that these inclusion bodies may contain lipids.

A draft of the genome of *E. cloacae* JD6301 was generated at the DOE Joint Genome Institute (JGI) using Illumina HiSeq 2000 technology. To remove any artifacts following Illumina sequencing, raw data were passed through DUK, a program developed by JGI. The Illumina reads were assembled using Velvet version 1.104 (2), and 1- to 3-kb simulated paired ends were created from Velvet contigs using wgsim (https://github.com/lh3/wgsim); the Illumina reads were then assembled with simulated pairs using Allpaths-LG versus r42328 (3). The assembly yielded 53 contigs in 49 scaffolds. For genome annotation, Prodigal was used to identify genes (4). This was followed by manual curation using GenePRIMP (5). tRNAScanSE (6) was used to find tRNA genes, while rRNA genes were identified using SILVA (7). INFERNAL (http://infernal.janelia.org) was used to identify noncoding RNAs. Other gene predictions and manual function annotation were performed using the Integrated Microbial Genomes (IMG) platform (http://img.jgi.doe.gov) developed by JGI (8).

The completed genome of strain JD6301 is 4,772,910 bp in length. There are an estimated 4,288,696 coding bases, and the G+C content is near 53%. There are a total of 4,509 protein-coding genes, with 84.91% having predicted functions. Of the genes identified, 4,246 match those of *E. cloacae*. Multiple multidrug efflux pumps were identified, which is common among *E. cloacae* strains (1). Genes associated with a type IV secretion system were also identified, another common feature of *E. cloacae* (9). Proteins associated with pilus production were also identified, which may have contributed to the inclusion bodies observed in this novel strain. The novel aspect of an *Enterobacter* sp. strain producing large quantities of lipids warrants further investigation.

### Nucleotide sequence accession numbers.

This draft genome sequence has been deposited in the IMG system under accession no. 20133 and GenBank under accession no. JDWH00000000.

## References

[1] Ren Y Ren Y Zhou Z Guo X Li Y Feng L 2010 Complete genome sequence of *Enterobacter cloacae* subsp. *cloacae* type strain ATCC 13047. J. Bacteriol. 192:2463–2464. 10.1128/JB.00067-10 20207761 PMC2863489

[2] Zerbino DR Birney E 2008 Velvet: algorithms for *de novo* short read assembly using de Bruijn graphs. Genome Res. 18:821–829. 10.1101/gr.074492.107 18349386 PMC2336801

[3] Gnerre S Maccallum I Przybylski D Ribeiro FJ Burton JN Walker BJ Sharpe T Hall G Shea TP Sykes S Berlin AM Aird D Costello M Daza R Williams L Nicol R Gnirke A Nusbaum C Lander ES Jaffe DB 2011 High-quality draft assemblies of mammalian genomes from massively parallel sequence data. Proc. Natl. Acad. Sci. U. S. A. 108:1513–1518. 10.1073/pnas.1017351108 21187386 PMC3029755

[4] Hyatt D Chen GL Locascio PF Land ML Larimer FW Hauser LJ 2010 Prodigal: prokaryotic gene recognition and translation initiation site identification. BMC Bioinformatics 11:119. 10.1186/1471-2105-11-119 20211023 PMC2848648

[5] Pati A Ivanova NN Mikhailova N Ovchinnikova G Hooper SD Lykidis A Kyrpides NC 2010 GenePRIMP: a gene prediction improvement pipeline for prokaryotic genomes. Nat. Methods 7:455–457. 10.1038/nmeth.1457 20436475

[6] Lowe TM Eddy SR 1997 tRNAscan-SE: a program for improved detection of transfer RNA genes in genomic sequence. Nucleic Acids Res. 25:955–964. 10.1093/nar/25.5.0955 9023104 PMC146525

[7] Pruesse E Quast C Knittel K Fuchs BM Ludwig W Peplies J Glockner FO 2007 Silva: a comprehensive online resource for quality checked and aligned ribosomal RNA sequence data compatible with ARB. Nucleic Acids Res. 35:7188–7196. 10.1093/nar/gkm864 17947321 PMC2175337

[8] Markowitz VM Mavromatis K Ivanova NN Chen IM Chu K Kyrpides NC 2009 IMG ER: a system for microbial genome annotation expert review and curation. Bioinformatics 25:2271–2278. 10.1093/bioinformatics/btp393 19561336

[9] Liu WY Wong CF Chung KM Jiang JW Leung FC 2013 Comparative genome analysis of *Enterobacter cloacae*. PLoS One 8:e74487. 10.1371/journal.pone.0074487 24069314 PMC3771936

